# Conducting research in clinical psychology practice: Barriers, facilitators, and recommendations

**DOI:** 10.1111/bjc.12142

**Published:** 2017-06-01

**Authors:** Kirsten V. Smith, Graham R. Thew

**Affiliations:** ^1^ Oxford Centre for Anxiety Disorders and Trauma Department of Experimental Psychology University of Oxford UK

**Keywords:** clinical psychology, research, research in practice, barriers to research, clinical psychology training, professional issues

## Abstract

**Objectives:**

The combination of clinical psychologists’ therapeutic expertise and research training means that they are in an ideal position to be conducting high‐quality research projects. However, despite these skills and the documented benefits of research to services and service users, research activity in practice remains low. This article aims to give an overview of the advantages of, and difficulties in conducting research in clinical practice.

**Method:**

We reviewed the relevant literature on barriers to research and reflected on our clinical and research experiences in a range of contexts to offer practical recommendations.

**Results:**

We considered factors involved in the planning, sourcing support, implementation, and dissemination phases of research, and outline suggestions to improve the feasibility of research projects in post‐qualification roles.

**Conclusions:**

We suggest that research leadership is particularly important within clinical psychology to ensure the profession's continued visibility and influence within health settings.

**Practitioner points:**

***Clinical implications***

- Emerging evidence suggests that clinical settings that foster research are associated with better patient outcomes.
- Suggestions to increase the feasibility of research projects in clinical settings are detailed.

***Limitations***

- The present recommendations are drawn from the authors’ practical experience and may need adaptation to individual practitioners’ settings.
- This study does not attempt to assess the efficacy of the strategies suggested.

## Background

There is a growing body of evidence that conducting research in clinical practice not only improves the clinical performance of the service (Mckeon *et al*., 2013) but can also lead to improved physical health outcomes and survival rates (Nickerson *et al*., 2014; Ozdemir *et al*., 2015; Rochon, du Bois, & Lange, 2014). Clinical psychologists in the United Kingdom are predominantly trained in the ‘scientist‐practitioner model’ meaning that we theoretically have the skills to both deliver psychological therapies and design, conduct, analyse, and interpret research (Holttum & Goble, 2006; Stricker, 2002). However, despite research output being a requirement of doctoral training, psychological research conducted in clinical practice post‐qualification is not commonplace (Mitchell & Gill, 2014; Morton, Patel, & Parker, 2008). In fact, it has been suggested that the modal number of publications for clinical psychologists, namely zero, has not improved in over twenty years (Barrom, Shadish, & Montgomery, 1988; Eke, Holttum, & Hayward, 2012; Norcross, Karpiak, & Santoro, 2005).

Clinical psychology trainees are required to produce a substantial and original piece of clinically‐relevant research as part of their training qualification. However, reports suggest that up to 75% of UK doctoral theses are left unpublished (Cooper & Turpin, 2007). One suggestion for these low publication rates is the lack of identification with the role of ‘researcher’ and rejection of the scientist‐practitioner model (Gelso, 1993; Newman & McKenzie, 2011). However, it seems important to broaden the conceptualization of the term ‘research activity’ to more than the production of peer‐reviewed publications and to include consuming research (e.g., reading literature, reviewing guidelines, staying up to date with recent field advances). While not falling under the formal definition of research, service evaluation (designed and conducted solely to define or judge current care) and audit (designed and conducted to inform delivery of best care by comparing current care against a predefined standard) could also reasonably constitute research activity given that they draw on similar skills (NHS Health Research Authority, 2014; see Table 1 for an overview of research types, their practical requirements, and general aims). Yet it has been suggested that even service evaluation and audit are not projects that clinical psychologists feel particularly comfortable undertaking (Cooper & Graham, 2009).

**Table 1:** Types of research activity in clinical psychology, requirements, aims, and potential team member involvement

Type of research	Practical requirements	General aim	Team members
Audit	Ongoing or fixed‐period data collection about an aspect of routine clinical practice	To assess whether current clinical practice is meeting a predetermined standard	C, M, T, J, A
Service evaluation/improvement	Fixed‐period data collection about an aspect of current clinical practice	To evaluate current clinical practice with a view to making improvements	C, M, T, J, A
Literature reviews	Searching, reading, and synthesizing existing data on a given topic	To summarize current literature and consider directions for further research	C, T, J
Meta‐analysis/synthesis	Combining and analysing data from multiple existing studies	To examine common research questions by pooling data from multiple sources	C, E, S
Case studies/series^a^	Conducting and describing a piece of clinical work with a person, group, or service, or a series of similar interventions	To describe clinical work that may be of interest to others (e.g., due to client presentation, method used, clinical reflection) and inform future clinical practice	C, T
Single‐case experimental designs^b^	Conducting and evaluating a piece of clinical work with a person, group, or service	To compare differences in an outcome before and after an intervention	C, T
Qualitative designs^b^	Obtaining and analysing interview, discourse, or written data from participants	To explore participants’ understandings and experiences	C, T, J
Experimental designs^b^	Research participants completing a fixed study paradigm	To evaluate the effect of manipulating a variable(s) on a particular outcome	C, T, J
Survey/questionnaire designs^b^	Research participants completing questionnaires	To explore the prevalence and range of participants’ responses on a given topic	C, T, J
Effectiveness studies^b^	Delivering and monitoring the effects of an intervention on participants in routine clinical settings	To examine the effectiveness of an intervention when delivered in a routine clinical context	C, M, T, J, A
RCTs^b^	Delivering and monitoring the effects of active or control interventions on participants under controlled conditions	To examine the efficacy of an intervention compared to a control group	C, T, J, A, E, S

Notes.

C = clinicians; M = managers; T = trainees; J = junior staff (e.g., assistant psychologists, research assistants, other junior staff members); A = administrative staff; E = external collaborators; S = statistical advisors; RCT = randomized controlled trial.

a Ethical review may be required.

b Ethical review required

A recent study showed that Australian psychologists working in a large metropolitan public health setting reported higher perceived capacity to undertake research compared with other allied health professionals (Elphinston & Pager, 2015). However, psychologists also perceived their individual capacity to be greater than that of their team and overarching organization, which may suggest they do not feel research skills are sufficiently valued or harnessed by employers. Perhaps unsurprisingly, team research capacity was found to mediate the relationship between psychologists’ research skills and their current research activity. Consequently, psychologists working in teams where research training was encouraged, funds were allocated, and projects relevant to practice were supported were more likely to engage with research and employ their skills. This study is consistent with earlier research that found subjective norms (i.e., beliefs about how others would perceive ones’ engagement in research) to be an important mediator between research environment and research intention (Eke *et al*., 2012; Holttum & Goble, 2006). Similarly, these findings were supported by a report on attitudes to research activity within the health and social care system in Ireland. This found that a lack of perceived skills, coupled with an organizational culture that did not value research, contributed to low research engagement (McHugh & Byrne, 2011). This underutilization of research training is troubling as it remains a unique selling point of clinical psychologists and an opportunity to provide intellectual leadership and influence policy within the health care profession. One explanation might be that, in the United Kingdom at least, research in clinical practice is so rare that there is limited opportunity for the benefits to be realized by wider teams, perhaps feeding the undervaluation of these skills.

However, this only partly explains the low research output of psychologists in clinical practice and unfortunately there is limited literature exploring the reasons for this. Some suggested barriers by McHugh and Byrne (2011) include the prioritization of clinical roles, lack of protected time, and lack of appropriate funding. In fact, over 80% of their participants cited either a lack of time or clinical work pressures as a factor preventing research activity. A recent report found that much of the research conducted within the National Health Service (NHS) was unfunded (Mitchell & Gill, 2014) with previous reports in the United States suggesting that as much as 40% of all research is carried out without adequate funding (Silberman & Snyderman, 1997), with 60% of unfunded projects being carried out in researchers’ own time (Schroter, Tite, & Kassem, 2006). Previous research found that competence in applying for funding was rated by both research active and inactive health professionals as their weakest skill. The authors suggested that insufficient practical experience due to limited funding opportunities may compound the lack of skill development (McHugh & Byrne, 2011). So it seems that we, as clinicians, have the difficult task of fitting research into limited time, with limited funds, often without the support or encouragement of our surrounding teams. Yet, as already mentioned, our research capacity enhances our professional visibility and influence within the field, as well as improving clinical performance and health outcomes.

In the light of the numerous benefits and difficulties, it is important to consider strategies that may facilitate research activity. We reflected on our clinical and research experiences in a range of contexts, aiming to outline key factors that can influence the successful set‐up and implementation of research in clinical practice. Relevant literature was consulted to consider the empirical support for these factors and to guide recommendations to overcome potential barriers.

## Determinants of successful research – Recommendations from the field

### Role specification

One factor that can make research projects easier to implement is having them ‘built in’ to overall job roles. Where psychologists are looking for post‐qualification positions or looking to change posts, it is worth considering how research components or specific projects fall within job descriptions. Having research activity included within the overall framework of roles and responsibilities for a post not only facilitates it happening, but also demonstrates something of the service's attitude towards this aspect of clinical psychologists’ skills. Where research is not mentioned, we recommend asking about what opportunities might be available, as it is likely this may be feasible at least in some form. If psychologists are enquiring more routinely about research opportunities within posts, this may contribute to research skills being more widely recognized as a key component of what the profession can offer.

We note that this approach does not apply exclusively to those seeking new posts, and would encourage psychologists to consider research opportunities within the context of job planning meetings, writing job descriptions for vacant posts, or annual appraisals.

### Scope of research project

A related point is the choice of research project itself. The size and setting of the service may mean larger‐scale studies are not practical in terms of resources, and original research studies will require formal ethical review,^1^ which can be lengthy, so may be a less practical option. However, in our experience the projects that are most difficult to implement in routine clinical settings are those where the impact of the findings may not be immediately apparent. While many may argue that the development of new knowledge is inherently valuable, clinical services must balance a number of competing priorities, meaning they can only feasibly support projects that are likely to lead directly to improved service provision and/or service user benefit. As such, it is recommended that clinicians in the first instance design research projects on the basis of client needs, and/or those with a greater focus on service improvement, which are more likely to be supported by services and clinical teams.

### Managerial support

In our experience, research projects in qualified practice hinge greatly on managerial support. Having team leaders, ward managers, or heads of service engaged with the project appears to make them much more feasible, especially in the planning and development stages. We would encourage clinicians to approach managers at the outset of a research project, and to elicit their ideas, interests, and priorities to help shape the project and foster further collaboration. Carving out adequate time for research may be a delicate subject to discuss with managers, especially with a busy caseload and clinical responsibilities. However, given the benefits outlined earlier, and it being among the core skills of our profession, we would encourage decisive advocacy for protected research time.

Previous research has shown that in a sample of research‐active health professionals in the North West of Ireland, almost half (45%) reported having to conduct their research mostly or completely outside of working hours (Research and Education Foundation, 2004). We would argue that it is not a reasonable expectation that research activity be subsumed into a schedule already at capacity, and doing this carries the risk of devaluing these skills within our profession. Therefore, we would suggest that managers are given a clear summary of the project, which should include (1) a description of the current problem or unknown issue, and possible implications of this; (2) a summary of potential benefits to service users, and the wider service if the project is done; (3) details of what methods will be used, including what time and resources are required, preferably with minimal impact on routine service provision; and (4) a timeline for the project and dissemination.

### Making the most of research time

Finding the time to undertake research projects in the context of a busy clinical service is not straightforward. While we acknowledge that this can be hard to implement, as far as is practical we strongly recommend aiming to designate particular blocks of time in which to undertake research activities, and have found that an effective method to protect this time is to work elsewhere if possible. This helps to keep the research time more distinct and serves as a more concrete reminder of this for both the researcher and other staff members. This approach can also minimize distractions and interruptions, which can reduce perceived effectiveness (Kearns & Gardiner, 2007). Clinicians may also want to consider the use of tools such as shared calendars, which can further clarify to the wider team when research time has been allocated. If practical, having this scheduled on a fixed, regular day and time can help make research activity become a more established routine within the service.

It should be noted that it is not necessarily the case that psychologists’ research activity is fully separate from their clinical responsibilities. Some research projects, such as case studies or case series, service user interviews, or single‐case experimental designs, have much greater integration with routine clinical service provision and will therefore require less ‘distinct’ research time (e.g., see Kaur, Murphy, & Smith, 2016; Ladd, Luiselli, & Baker, 2009; Thew & Krohnert, 2015).

### Project marketing

Research projects in clinical contexts will require a certain degree of marketing. Having sought and hopefully obtained managerial support, it is helpful to publicize the project, for example, through in‐house presentations, discussion with service users, and service newsletters, magazines, or social media accounts. We have found that projects benefit greatly from the extra visibility and, to some extent, legitimacy that this provides. The marketing approach needs to extend throughout the project to maintain this visibility, which can be achieved through giving brief updates on the status of the project, and taking the time to feed back the results, particularly to staff who may have been involved with recruiting participants or in other capacities. This is also critical to influencing the culture of a service to be more receptive to future research projects.

### Funding

Some, although not all, projects will require at least some funding, for example, to purchase equipment or resources, to buy out part of a clinician's time, or to recruit a research assistant, and preparing a successful funding application in this competitive climate can be time‐intensive. While this can understandably be a barrier to research activity in some contexts, we would emphasize that funding is by no means required for a successful research project, particularly when there is interest and support from the immediate clinical team including assistants and trainees, or a skilled wider network.

Where funding is being sought, we note that a number of services and trusts have some funds available to support new research projects, particularly those looking to innovate, or deliver more effective and efficient interventions for service users. We recommend working closely with local Research and Development departments, who are able to advise on funding opportunities, and on various aspects of developing and running projects generally. Many charitable organizations fund psychologist‐led projects (examples include the following: MQ: Transforming Mental Health through Research; British Heart Foundation; Marie Curie Cancer Care; Mind; OCD‐UK). At a broader level, agencies such as the National Institute for Health Research, the Wellcome Trust, and the Alzheimer's Society offer more structured programmes of funding to support clinicians in undertaking research projects linked to a clinical or academic institution.

### Collaboration

While the research projects conducted as part of clinical training courses tend to be solo efforts with a small number of supervisors, post‐qualification research is able to place a greater emphasis on collaboration. This could be within or across services, and links between clinical services and academic institutions can often be productive. Here, clinicians can benefit from academics’ research expertise and supervision, while academics can benefit from clinicians’ practical experience and knowledge, along with potential links to service users interested in contributing to research studies (Lampropoulos *et al*., 2002). For example, involvement with academic departments could permit the independent evaluation of local clinical services and establish a protocol and methods for ongoing data collection. On a smaller scale, potential collaborations could include supervising the research projects of clinical trainees and postgraduate junior academics. While collaborations will help to reduce the demands on an individual researcher, they also can serve to maintain the momentum of a research project given multiple people are invested in its completion.

It may also be the case that individuals are willing to assist with the project in a more informal capacity, such as helping with recruitment or general administration. It can be helpful to discuss in the early stages of projects the level of involvement different collaborators will have, and to work out the practical elements of how best to keep people informed and updated with what they might be required to do.

### Deadlines and monitoring progress

Although there may be an estimated timescale for the project agreed at the outset, we have found that setting deadlines for different stages of the project can help maintain progress, and prevent the project being overshadowed or neglected in the face of new service‐level priorities or responsibilities. Obviously, a degree of flexibility will always be required, but working to an agreed schedule, and if possible having someone who is more external to the project monitoring its progress, such as a manager or mentor, can be helpful.

### Dissemination

Dissemination of project findings can often be a somewhat neglected part of the research process (Cooper & Turpin, 2007), but it can play a powerful role in facilitating subsequent service improvements, research projects, and future funding applications. Failing to share and publicize project findings can mean people are unable to see their value and implications, which can therefore hinder research projects from happening in the future.

Publication in peer‐reviewed journals is one effective route to share findings, but there are many others that should also be considered, including presenting at conferences, at team meetings, or directly to service leaders, service users, project participants, and where relevant, to those managing or funding services. To maximize dissemination effectiveness, it will be necessary to adapt the medium and language of your communications to suit a range of audiences. It may be possible to circulate written summaries or brief reports around local or regional professional networks and industry partnerships, and again making use of in‐house media/communication teams can facilitate this.

We note that for some larger projects, the time and effort invested in just obtaining the results can be significant, meaning that finding further time and/or motivation to apply to dissemination activities can be difficult. However, it can be argued that given most projects involve collecting data from participants in some form, who have therefore given their time and energy to assist with the aims of the project, we have a professional duty to make productive use of the findings and ensure that they are shared appropriately. We recommend including dissemination activity in the project timeline from the outset to avoid this being neglected or missed.

### Feeling deskilled

Lastly, it is worth noting that for many psychologists, the idea of developing a research project may feel demanding or even daunting and that this may be the principal reason that research ideas do not get taken forward (Cooper & Graham, 2009). It is easy to feel that our research skills are no longer up to date, or that our projects will require too much time to be feasible.

Given that this may understandably encourage avoidance of research activity and that as psychologists we all recognize that avoidant strategies are not the most useful in the long term, we have found it helpful to remember the following: First, research projects do not have to use complicated methodology and large samples in order to have scientific merit and useful implications. Second, research activity can be quite closely tied into routine clinical work as described earlier. Third, seeking out potential continuing professional development (CPD) opportunities through workshops, conference attendance, and training activities can improve research skills and increase confidence. Fourth, no researcher knows how to do everything, and that collaboration can be a powerful tool for learning new skills, and lastly, psychologists already have a number of transferable skills from their clinical work, such as the ability to approach a problem logically and systematically, or the capacity to attend accurately and consider carefully what a client is saying, which are equally important and valuable within the research domain.

## Conclusions

Despite a strong focus on research skills during clinical psychologists’ training, the evidence suggests that post‐qualification research activity within clinical settings is rare, even though there are tangible benefits to clients and services. While a lack of time to undertake research within clinical roles is perhaps the most obvious reason for this, we have outlined a number of other possible barriers and hope that some of our reflections and suggestions may prove useful to those clinicians who are considering undertaking research projects within their services.

Clinical psychologists’ combination of clinical expertise and research training means that they are in an ideal position to be conducting high‐quality research projects that aim to better understand and intervene across a range of clinical issues. From a professional perspective, these research skills are perhaps one of the key features of clinical psychologists that serve to distinguish us from many health professionals. In a context of financial pressures and cuts to clinical services and training places, it is possible that greater use of these research skills in practice will help to ensure the continued appeal and future utility of clinical psychology.

## Note

1 Whether ethical review is required depends on the nature of the project and the participants involved. Ethical approval should be sought through the Integrated Research Application System (NHS REC), university review boards, or the Social Care Research Ethics Committee. In the case of independent practice where practitioners may not have access to an ethical review process, they should be able to demonstrate that they have adhered to the *Code of Ethics and Conduct* and the *Code of Human Research Ethics* outlined by the British Psychological Society (British Psychological Society, 2006, 2014).
